# The *In Vitro* Antioxidant Activity and Inhibition of Intracellular Reactive Oxygen Species of Sweet Potato Leaf Polyphenols

**DOI:** 10.1155/2018/9017828

**Published:** 2018-02-15

**Authors:** Hongnan Sun, Bona Mu, Zhen Song, Zhimin Ma, Taihua Mu

**Affiliations:** ^1^Key Laboratory of Agro-products Processing, Ministry of Agriculture, Laboratory of Food Chemistry and Nutrition Science, Institute of Food Science and Technology, Chinese Academy of Agricultural Sciences, 2 Yuan Ming Yuan West Road, Haidian District, Beijing 100193, China; ^2^Department of Crop Genomics and Genetic Improvement, Biotechnology Research Institute, Chinese Academy of Agricultural Sciences, 12 Zhongguancun South Street, Haidian District, Beijing 100081, China; ^3^Institute of Cereal and Oil Crops, Key Laboratory of Crop Genetics and Breeding of Hebei Academy of Agriculture and Forestry Sciences, 162 Heng Shan Road, Gaoxin District, Shijiazhuang 050035, China

## Abstract

The *in vitro* antioxidant activity and inhibition of intracellular reactive oxygen species (ROS) of the total and individual phenolic compounds from Yuzi No. 7 sweet potato leaves were investigated in this study. Sweet potato leaf polyphenols possessed significantly higher antioxidant activity than ascorbic acid, tea polyphenols, and grape seed polyphenols. Among the individual phenolic compounds, caffeic acid showed the highest antioxidant activity, followed by monocaffeoylquinic acids and dicaffeoylquinic acids, while 3,4,5-tri-O-caffeoylquinic acid showed the lowest value. Sweet potato leaf polyphenols could significantly decrease the level of intracellular ROS in a dose-dependent manner. The order of the inhibiting effect of individual phenolic compounds on the intracellular ROS level was not in accordance with that of antioxidant activity, suggesting that there was no direct relationship between antioxidant activity and intracellular ROS-inhibiting effect. Sweet potato leaves could be a good source of biologically active polyphenols with multiple applications in the development of foods, health products, pharmaceuticals, and cosmetics.

## 1. Introduction

Reactive oxygen species (ROS) are a series of metabolic byproducts involved in degenerative and pathological processes in the human body [1]. Overproduction of ROS could disturb cellular redox balance, resulting in cell injury or apoptosis [2], further triggering oxidative damage of tissues and organs, which accelerates the development of various diseases, such as cancer, atherosclerosis, diabetes, chronic inflammatory disease, cardiovascular disease, and Alzheimer [3–6]. Although humans and other organisms have endogenous antioxidant defenses against ROS, these systems may sometimes not be sufficient to prevent the occurrence of cell damage [7].

Synthetic antioxidants are widely used in the food industry, to prevent the production of toxicities or mutagenicities that may present health hazards [8]. However, studies have shown that synthetic antioxidants have chemical toxicity, which can increase the risk of cancer and damage the liver [9–12]. Consequently, it is particularly important to look for natural antioxidants that can replace synthetic antioxidants. One of the natural antioxidants, plant polyphenols, which are widely found in fruits and vegetables, is known as potent antioxidant and free radial scavenger, and thus possesses many biological activities, such as antioxidation, antiaging, and prevention of cardiovascular disease, cancer inhibition, anti-inflammatory effects, antiviral effects, antibacterial effects, and so forth [13–17]. That is to say, plant polyphenols have the potential to be widely used as natural antioxidants in food, cosmetic, pharmaceutical, and medicinal products [18].

Sweet potato leaves are the aboveground parts of sweet potato (*Ipomoea batatas* L.), which can be harvested several times a year, but most of the sweet potato leaves in China have been discarded or used as feed, causing serious environmental pollution and waste of resources [19]. In our previous study, it has been found that sweet potato leaves are rich in polyphenols, with the content ranging from 2.73 to 12.46 g/100 g dry weight (DW) [20, 21]. Meanwhile, the *in vitro* antioxidant activity of polyphenols extracted and purified from sweet potato leaves (cultivars: Yuzi No. 7 and Ximeng No. 1) was also investigated in our previous study, and the results showed that sweet potato leaf polyphenols possessed strong *in vitro* antioxidant activity, which is 2 times higher than ascorbic acid, tea polyphenols, and grape seed polyphenols [19]. The above-mentioned results indicate that sweet potato leaf polyphenols have great potential to be widely used in food, healthcare, pharmaceutical, and cosmetic industries. However, the study on sweet potato leaf polyphenols is just in its early stages. Most studies have focused on the extraction and purification of polyphenols from sweet potato leaves and the antioxidant activity *in vitro* [19, 22]. So far, no studies were undertaken to explore the inhibition of intracellular ROS.

Our previous study showed that the correlation coefficient between antioxidant activities of sweet potato leaves and polyphenols were the highest, followed by carbohydrate. There were negative correlation coefficients between antioxidant activity and protein, fat, and crude fiber [20, 21]. In addition, the antioxidant activity of polyphenols is largely up to their individual phenolic compound composition [19]. Iwai et al. [23] found that the antioxidant activity of dicaffeoylquinic acid was 2 times higher than that of monocaffeoylquinic acids. Our previous study showed that sweet potato leaf polyphenols were mainly composed of seven caffeoylquinic acids and a small amount of caffeic acid [19], which was similar to other researchers' reports [24–26]. However, there is little information about the contribution rate of different individual phenolic compounds from sweet potato leaves on the *in vitro* antioxidant activity and the inhibition of intracellular ROS.

Therefore, in the present study, photochemiluminescence (PCL) assay and oxygen radical absorbance capacity (ORAC) method were used to analyze the *in vitro* antioxidant activity of sweet potato leaf polyphenols and their individual phenolic compounds. Furthermore, H_2_O_2_ was used to induce and establish human hepatocyte LO2 oxidative stress model, and the inhibition of intracellular reactive oxygen species of sweet potato leaf polyphenols and their individual phenolic compounds were investigated. The purpose is to make the *in vitro* antioxidant activity and inhibition of intracellular reactive oxygen species of sweet potato leaf polyphenols clear and to further lay a theoretical foundation for the development and utilization of sweet potato leaf polyphenols.

## 2. Materials and Methods

### 2.1. Materials

Sweet potato leaf variety, Yuzi No. 7 (which was bred by hybridization, not a genetically modified organism), was collected from the Research Institute of Sweet Potato of the Chinese Academy of Agricultural Sciences (Xuzhou, China), which was planted with standard production practices in early March, 2015, and collected in the middle of August. Then, sweet potato leaves were washed, freeze dried, ground, and stored at 4°C in sealed aluminum foil bags for further use. The proximate composition and total polyphenol content (TPC, measured by Folin-Ciocalteu method [27, 28]) of Yuzi No. 7 sweet potato leaves were as follows: moisture 88.16 ± 0.51 g/100 g fresh weight, protein 22.09 ± 0.34 g/100 g dry weight (DW), fat 2.36 ± 0.07 g/100 g DW, dietary fiber 36.52 ± 0.75 g/100 g DW, carbohydrate 50.22 ± 0.85 g/100 g DW, gross energy 421.39 ± 1.05 kcal/100 g DW, ash 8.91 ± 0.76 g/100 g DW, and TPC 12.97 ± 0.82 g chlorogenic acid equivalent (CAE)/100 g DW.

Then, the extraction of polyphenols from sweet potato leaves was carried out according to the method described by Sun et al. [20, 21]), and the purification of crude polyphenol extract was carried out according to the method established by Xi et al. [19] and Sun et al. [27]. TPC of sweet potato leaf polyphenols after being purified by AB-8 resin was 87.56 ± 1.38%.

### 2.2. Reagents

Folin-Ciocalteu reagent, 2,2′-azobis (2-amidinopropane) dihydrochloride (AAPH), ascorbic acid, 2,5,7,8-tetramethylchroman-2-carboxylic acid (Trolox), chlorogenic acid, caffeic acid, 2′,7′-dichlorofluorescein diacetate (DCFDA),McCoy's 5A medium, methyl thiazolyl tetrazolium (MTT), dimethyl sulfoxide (DMSO), crystal violet, and chromatography grade acetonitrile and methanol were purchased from Sigma-Aldrich Inc. (St. Louis, MO, USA). The chromatography grade caffeoylquinic acid standards (3-O-caffeoylquinic acid, 4-O-caffeoylquinic acid, 5-O-caffeoylquinic acid, 3,4-di-O-caffeoylquinic acid, 3,5-di-O-caffeoylquinic acid, 4,5-di-O-caffeoylquinic acid, and 3,4,5-tri-O-caffeoylquinic acid) were purchased from AMRESCO Biotechnology Co. Ltd. (Solon, OH, USA). Tea polyphenols (TP) and grape seed polyphenols (GSP) were purchased from Yihe Biotechnology Co. Ltd., Xi'an, China. Fetal bovine serum (FBS) was purchased from GE Healthcare Life Sciences HyClone Laboratories (Logan, Utah, USA). Penicillin/streptomycin was purchased from Mediatech Inc. (Manassas, Virginia, USA). Trypsin (1 : 250, activity: 250 NFU/mg) was purchased from BioD BioTech Co. Ltd. (Beijing, China). Sodium fluorescein, sodium hydroxide, phosphate, and other analytical grade reagents were purchased from Beijing Chemical Reagents Co. (Beijing, China).

### 2.3. Extraction of Polyphenols from Sweet Potato Leaves

Extraction of polyphenols from sweet potato leaves was carried out according to the method described by Sun et al. [20, 21]). Briefly, 10 g of leaf powder was extracted with 200 mL of 70% (*v*/*v*) ethanol for 30 min at 50°C and subjected to ultrasonic wave treatment (59 kHz). Following centrifugation at 5000*g* for 10 min at 4°C, the residue was reextracted twice with 70% ethanol as described above. The supernatants were pooled, concentrated in a rotary evaporator at 45°C, and freeze dried to obtain a crude polyphenol extract.

### 2.4. Total Polyphenol Content

Total polyphenol content (TPC) was measured by the Folin-Ciocalteu method [27, 28]. The crude extract was dissolved in 100 mL distilled water; an aliquot (0.5 mL) was mixed with 1.0 mL of Folin-Ciocalteu reagent, previously diluted 10 times, and allowed to react at 30°C for 30 min. Subsequently, 2.0 mL of saturated Na_2_CO_3_ (10%, *w*/*v*) was added to the mixture. The following 30 min, absorbance was measured at 736 nm in a UV1101 spectrophotometer (Hitachi, Japan). A calibration curve consisting of chlorogenic acid standards, ranging from 0.02 to 0.10 mg/mL, was prepared. TPC was expressed as chlorogenic acid equivalent (CAE) on a dry weight (DW) basis. TPC of Yuzi No. 7 sweet potato leaves was 12.97 ± 0.82 g CAE/100 g DW.

### 2.5. Purification of Polyphenols from Sweet Potato Leaves by AB-8 Macroporous Resins

The purification of crude polyphenol extract was carried out according to the method established by Xi et al. [19] and Sun et al. [27]. Briefly, the crude polyphenol extract was dissolved in distilled water to get a crude polyphenol solution of 2.0 mg CAE/mL and adjusted to pH 3.0 using 2.0 mol/L HCl. The purification process was carried out in a glass column (1 cm × 10 cm) wet packed with pretreated AB-8 resin. The bed volume (BV) of the resins was 10 mL (equal to 5 g resin). The crude polyphenol solution was allowed to flow through the glass column at a flow rate of 1.0 mL/min (the volume ratio between crude polyphenol solution and BV was 5 : 1). After the adsorption equilibrium had been reached, the column was first washed with distilled water at a flow rate of 1.0 mL/min until the effluent was clear and then eluted by 70% (*v*/*v*) ethanol solution at a flow rate of 1.0 mL/min (the volume ratio between ethanol solution and BV was 3 : 1). The eluted solution was collected and concentrated in a rotary evaporator at 45°C to remove the ethanol and then freeze dried. The total polyphenol content of purified polyphenols from sweet potato leaves was 87.56 ± 1.38%.

### 2.6. Quantification of Individual Phenolic Compounds by Reversed-Phase HPLC (RP-HPLC)

Individual phenolic compounds in polyphenols from sweet potato leaves were evaluated by RP-HPLC (Agilent Technologies, Palo Alto, CA, USA) according to the method described by Sun et al. [27]. Spectral data from 200 to 800 nm were recorded, and the polyphenol chromatogram was monitored at 326 nm. Caffeic acid, 3-O-caffeoylquinic acid, 4-O-caffeoylquinic acid, 5-O-caffeoylquinic acid, 3,4-di-O-caffeoylquinic acid, 3,5-di-O-caffeoylquinic acid, 4,5-di-O-caffeoylquinic acid, and 3,4,5-tri-O-caffeoylquinic acid were used as standard. Identification and quantitative analysis were done by comparison with standards. The amount of individual phenolic compound was expressed as g/100 g of purified sweet potato leaf polyphenols on a dry weight basis (g/100 g DW).

### 2.7. Antioxidant Activity Analysis

#### 2.7.1. Photochemiluminescence Assay

Photochemiluminescence assay was carried out using an automated photo chemiluminescent (PCL) system (Photochem, Analytik Jena AG, Germany), according to the method reported by Cofrades et al. [29]. Briefly, 20 *μ*L of sample solution at different concentrations (5, 10, and 20 *μ*g/mL) was used in a commercial kit for antioxidant capacity determination. Ascorbic acid was used as the standard. The results were expressed as ascorbic acid equivalent (ACE) relative to sample solution volume (*μ*g·ACE/mL).

#### 2.7.2. Oxygen Radical Absorbance Capacity Assay

Oxygen radical absorbance capacity (ORAC) assay was carried out according to the method described by Sun et al. [27]. Briefly, all samples and reagents in the experiment were dissolved and diluted with phosphate buffer (0.075 M, pH 7.4). 20 *μ*L sample solutions at different concentrations (5, 10, and 20 *μ*g/mL) were added to 20 *μ*L phosphate buffer and then mixed with 20 *μ*L·63 nmol/L sodium fluorescein solution in a clear 96-well microplate and incubated at 37°C for 15 min. Then, 140 *μ*L·18.28 mmol/L AAPH solution was rapidly added to the well. Fluorescence was read at 485 nm excitation and 535 nm emission until complete extinction. ORAC values were expressed as *μ*g·Trolox equivalent (TE)/mL (*μ*g·TE/mL).

### 2.8. Inhibition of Intracellular Reactive Oxygen Species of Sweet Potato Leaf Polyphenols

#### 2.8.1. Cell Culture

Human hepatocyte LO2 cells were obtained from Bioye Biological Technology Co. Ltd. (Shanghai, China). Cells were cultured in McCoy's 5A culture medium, containing 10% of FBS and 1% of penicillin/streptomycin, at 37°C in an atmosphere of 5% CO_2_ in air with controlled humidity.

#### 2.8.2. Determination of H_2_O_2_ Concentration in the Establishment of LO2 Oxidative Stress Model

The H_2_O_2_ stock solution was diluted with McCoy's 5A culture medium without FBS into H_2_O_2_ working solution of different concentrations: 1, 5, 25, 50, 100, 200, 400, 500, and 1000 *μ*M. LO2 cells were seeded into 96-well cell culture plates at a density of 5 × 10^3^ cells/well and then incubated for 24 h. After that, the culture medium in 96-well cell culture plates was discarded, and the cells were washed once with 200 *μ*L of PBS in each well. Subsequently, the H_2_O_2_ working solution of different concentrations was added to 96-well cell culture plates. Following incubation for another 6 h, the H_2_O_2_ working solution was discarded, and 100 *μ*L of FBS-free culture medium was added to each well; then, 20 *μ*L of 5.0 mg/mL MTT solution was added to each well. After incubation for another 4 h, the supernatant in each well was discarded. Then, the generated formazan precipitate was dissolved in 150 *μ*L of DMSO, and absorbance was measured at 570 nm using a RT-6000 microplate reader (Rayto, Shenzhen, China). The cell viability was calculated according to (1). The H_2_O_2_ concentration when the cell viability was reduced to 50% was chosen to be the optimal concentration to establish human hepatocyte LO2 oxidative stress model.

(1) $$\begin{array}{c} Cell\;viability\left(\%\right)=\frac{A_{n}}{A_{0}}\times 100, \end{array}$$

wherein A_n_ was the absorbance value of H_2_O_2_ treatment group and A_0_ was the absorbance value of blank control group.

#### 2.8.3. Determination of the Concentration Range of Polyphenols from Sweet Potato Leaves

The sweet potato leaf polyphenol stock solution was diluted with FBS-free McCoy's 5A culture medium into sweet potato leaf polyphenol working solution of different concentrations: 25, 50, 100, 200, 400, 800, 1000, 1500, 2000, and 3000 *μ*g/mL. LO2 cells were seeded into 96-well cell culture plates at a density of 5 × 10^3^ cells/well and then incubated for 24 h. After that, the culture medium in 96-well cell culture plates was discarded and the cells were washed once with 200 *μ*L of PBS in each well. Subsequently, the sweet potato leaf polyphenol working solution of different concentrations was added to 96-well cell culture plates. Following incubation for another 24 h, the sweet potato leaf polyphenol working solution was discarded and 100 *μ*L of FBS-free culture medium was added to each well; then, 20 *μ*L of 5.0 mg/mL MTT solution was added to each well. The cell viability test was carried out according to the protocol described in the last section.

#### 2.8.4. Effect of Sweet Potato Leaf Polyphenols on the Cell Viability of Oxidative Stress LO2 Cells

The experiment was divided into 7 groups: blank control group, oxidative stress model group, Trolox-pretreated oxidative stress model group, ascorbic acid-pretreated oxidative stress model group, tea polyphenol-pretreated oxidative stress model group, grape seed polyphenol-pretreated oxidative stress model group, and sweet potato leaf polyphenol-pretreated oxidative stress model group.

LO2 cells were seeded into 96-well cell culture plates at a density of 5 × 10^3^ cells/well and then incubated for 24 h. After that, the culture medium in 96-well cell culture plates was discarded and the cells were washed once with 200 *μ*L of PBS in each well. Subsequently, the sweet potato leaf polyphenol working solution of different concentrations/Trolox solution of a certain concentration/ascorbic acid solution of a certain concentration/tea polyphenols solution of a certain concentration/grape seed polyphenols solution of a certain concentration was added to 96-well cell culture plates. Following incubation for another 24 h, the culture medium was discarded and the cells were washed once with 200 *μ*L of PBS in each well. Then, H_2_O_2_ working solution of a certain concentration (which was used to establish LO2 oxidative stress model) was added to 96-well cell culture plates. Following incubation for another 6 h, the culture medium was discarded and 100 *μ*L of FBS-free culture medium was added to each well; then, 20 *μ*L of 5.0 mg/mL MTT solution was added to each well. The cell viability test was carried out according to the protocol described in the last section.

#### 2.8.5. Effect of Individual Phenolic Compounds from Sweet Potato Leaves on the Cell Viability of Oxidative Stress LO2 Cells

The experiment was divided into 10 groups: blank control group, oxidative stress model group, sweet potato leaf polyphenol-pretreated oxidative stress model group, 3-O-caffeoylquinic acid-pretreated oxidative stress model group, 4-O-caffeoylquinic acid-pretreated oxidative stress model group, 5-O-caffeoylquinic acid-pretreated oxidative stress model group, 3,4-di-O-caffeoylquinic acid-pretreated oxidative stress model group, 3,5-di-O-caffeoylquinic acid-pretreated oxidative stress model group, 4,5-di-O-caffeoylquinic acid-pretreated oxidative stress model group, and 3,4,5-tri-O-caffeoylquinic acid-pretreated oxidative stress model group. The experimental procedure was the same as described in the above paragraph.

#### 2.8.6. Effects of Sweet Potato Leaf Polyphenols on the Level of Intracellular Reactive Oxygen Species

The experiment was divided into 7 groups: blank control group, oxidative stress model group, Trolox-pretreated oxidative stress model group, ascorbic acid-pretreated oxidative stress model group, tea polyphenol-pretreated oxidative stress model group, grape seed polyphenol-pretreated oxidative stress model group, and sweet potato leaf polyphenol-pretreated oxidative stress model group.

LO2 cells were seeded into 96-well cell culture plates at a density of 5 × 10^3^ cells/well and then incubated for 24 h. After that, the culture medium in 96-well cell culture plates was discarded and the cells were washed once with 200 *μ*L of PBS in each well. Subsequently, the sweet potato leaf polyphenol working solution of different concentrations/Trolox solution of a certain concentration/ascorbic acid solution of a certain concentration/tea polyphenols solution of a certain concentration/grape seed polyphenols solution of a certain concentration was added to 96-well cell culture plates. Following incubation for another 24 h, the culture medium was discarded and the cells were washed once with 200 *μ*L of PBS in each well. 10 *μ*M of H_2_DCF-DA was added to each well. After incubation away from light for 30 min, the cells were washed once with 200 *μ*L of PBS in each well. Then, H_2_O_2_ working solution of a certain concentration (which was used to establish LO2 oxidative stress model) was added to 96-well cell culture plates. Following incubation away from light for another 30 min, the fluorescence intensity was read at 485 nm excitation and 530 nm emission using a microplate reader (Chameleon, Hidex, Turku, Finland). The level of intracellular reactive oxygen species was calculated as the following:

(2) $$\begin{array}{c} \text{The }level\text{ of }\mathrm{int}racellular\;reactive\;oxygen\;species\left(\%\right)=\frac{{FI}_{n}}{{FI}_{0}}\times 100, \end{array}$$

wherein FI_n_ is the fluorescence intensity of the pretreated groups and FI_0_ is the fluorescence intensity of the oxidative stress model group.

#### 2.8.7. Effects of Individual Phenolic Compounds from Sweet Potato Leaves on the Level of Intracellular Reactive Oxygen Species

The experiment was divided into 10 groups: blank control group, oxidative stress model group, sweet potato leaf polyphenol-pretreated oxidative stress model group, 3-O-caffeoylquinic acid-pretreated oxidative stress model group, 4-O-caffeoylquinic acid-pretreated oxidative stress model group, 5-O-caffeoylquinic acid-pretreated oxidative stress model group, 3,4-di-O-caffeoylquinic acid-pretreated oxidative stress model group, 3,5-di-O-caffeoylquinic acid-pretreated oxidative stress model group, 4,5-di-O-caffeoylquinic acid-pretreated oxidative stress model group, and 3,4,5-tri-O-caffeoylquinic acid-pretreated oxidative stress model group. The experimental procedure was the same as described in the above paragraph.

### 2.9. Statistical Analysis

All the above-mentioned experiments were performed for at least three replicates. The results were expressed as mean ± SD. Statistical analysis was carried out by means of one-way ANOVA followed by a Duncan multiple comparison test with the SAS version 8.1 software (SAS Institute Inc., Cary, NC, USA). Statistical significance was set at *p* < 0.05.

## 3. Results and Discussion

### 3.1. Quantification of Individual Phenolic Compounds by RP-HPLC

Table 1 showed that there were 7 caffeoylquinic acids and a small quantity of caffeic acid detected from Yuzi No. 7 sweet potato leaf polyphenols, which was in accordance with the previous reports [19–21, 24–26]. The three dicaffeoylquinic acids showed the highest contents, especially 3,5-di-O-caffeoylquinic acid (36.30 ± 0.19%, DW), followed by 3,4-di-O-caffeoylquinic acid (25.01 ± 0.42%, DW), 4,5-di-O-caffeoylquinic acid (21.38 ± 0.21%, DW), 3,4,5-tri-O-caffeoylquinic acid, monocaffeoylquinic acids, and caffeic acid. The composition of individual phenolic compounds in sweet potato leaves could be mainly attributed to the different genotypes and agroecological environment [30]. But in general, from the result obtained in this study regarding the composition of the individual phenolic compounds, sweet potato leaves could be a good source of biologically active compounds with multiple applications in the development of health products, functional foods, pharmaceuticals, and cosmetics. However, in pursuit of application in practical production, it is necessary to clarify the biological activities of each individual phenolic compound.

### 3.2. Antioxidant Activity

#### 3.2.1. Antioxidant Activity of Total Polyphenols from Sweet Potato Leaves

The antioxidant activity of total polyphenols from Yuzi No. 7 sweet potato leaves was shown in Table 2. The··O_2_^−^ scavenging activity showed significant dose dependence (*p* < 0.05). Under all tested concentrations, the··O_2_^−^ scavenging activity of total polyphenols from sweet potato leaves was higher than that of total polyphenols from tea and grape seeds. When the concentration reached 20 *μ*g/mL, total polyphenols from sweet potato leaves showed the highest··O_2_^−^ scavenging activity (62.71 *μ*g·ACE/mL), which was 4.90 and 8.32 times higher than that of total polyphenols from tea and grape seeds, respectively. The oxygen radical absorbance capacity of total polyphenols from sweet potato leaves also showed a significant dose-dependent relationship (*p* < 0.05). At the concentration of 20 *μ*g/mL, the oxygen radical absorbance capacity of total polyphenols from sweet potato leaves was the highest (55.68 *μ*g·TE/mL), which was 1.28 times higher than that of total polyphenols from tea and grape seeds.

#### 3.2.2. Antioxidant Activity of Individual Phenolic Compounds from Sweet Potato Leaves

The antioxidant activity of individual phenolic compounds from Yuzi No. 7 sweet potato leaves was shown in Table 3. For the ·O_2_^−^ scavenging activity, caffeic acid showed the highest value (51.12 *μ*g·ACE/mL), which was much higher than total polyphenols from sweet potato leaves (30.56 *μ*g·ACE/mL). However, the ·O_2_^−^ scavenging activities of all individual caffeoylquinic acids was lower than those of caffeic acid and total polyphenols from sweet potato leaves. Among which, the monocaffeoylquinic acids and dicaffeoylquinic acids showed no significantly different values and 3,4,5-tri-O-caffeoylquinic acid showed the lowest value (15.03 *μ*g·ACE/mL).

For the oxygen radical absorbance capacity, all the individual phenolic compounds except 3,4,5-CQA showed higher values than total polyphenols from sweet potato leaves (33.72 *μ*g·TE/mL). Caffeic acid showed the highest value (56.78 *μ*g·TE/mL), which was significantly higher than other individual phenolic compounds (*p* < 0.05). There was no significant difference among the oxygen radical absorbance capacity of monocaffeoylquinic acids and dicaffeoylquinic acids.

Iwai et al. [23] reported that the radical scavenging activity of caffeoylquinic acid derivatives was positively correlated with the number of caffeoyl groups in their molecules. However, in our present study, there was no significant difference among the antioxidant activities of monocaffeoylquinic acids and dicaffeoylquinic acids, which was not in accordance with the report of Iwai et al. One of the possible reasons was that the methods used in determining antioxidant activity were different. In other words, different free radical scavenging activities were detected in the study of Iwai et al. and ours, for example, DPPH radical scavenging activity was detected in the study of Iwai et al., while the ·O_2_^−^ scavenging activity and oxygen radical absorbance capacity were detected in our present study. Moreover, Bendary et al. [31] reported that the antioxidant activity of polyphenols was mainly related to the number of phenolic hydroxyl groups in the molecules—the higher the phenolic hydroxyl number, the stronger the antioxidant activity. The molecular weight of mono- and dicaffeoylquinic acids was 354.31 and 516.45, respectively, and the phenolic hydroxyl group number in mono- and dicaffeoylquinic acids was 5 and 6, respectively. That is to say, the number ratio of phenolic hydroxyl groups in the same concentration of mono- and dicaffeoylquinic acids in the present study was 1.21, which possessed little difference. Therefore, at the same mass concentration, the antioxidant activity was not significantly different between mono- and dicaffeoylquinic acids. In addition, the antioxidant activity of polyphenols was related not only to the number of phenolic hydroxyl groups but also to the position of phenolic hydroxyl groups [32]. The position of phenolic hydroxyl groups and spatial conformation of different caffeoylquinic acids were different, making it very difficult to judge which one possesses higher antioxidant activity.

Although caffeic acid possessed the highest antioxidant activity, its content in the total polyphenols from sweet potato leaves was only 0.09%. By contrast, the antioxidant activity of mono- and dicaffeoylquinic acids was lower than that of caffeic acid, but the content of dicaffeoylquinic acids was predominant, accounting for 82.69% of the total polyphenols in sweet potato leaves. It can therefore be said that dicaffeoylquinic acids contribute most to the antioxidant activity of sweet potato leaf polyphenols.

### 3.3. Inhibition of Intracellular Reactive Oxygen Species of Sweet Potato Leaf Polyphenols

#### 3.3.1. Determination of H_2_O_2_ Concentration in the Establishment of LO2 Oxidative Stress Model

**Figure 1:**
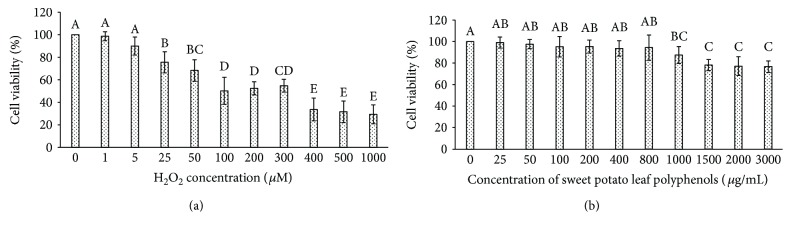
The effect of (a) H_2_O_2_ concentration and (b) sweet potato leaf polyphenol concentration on the cell viability of human hepatocyte LO2 cells. Values were means ± SD of five determinations. The different letters above the different bars mean that the cell viability was significantly different (*p* < 0.05).

MTT test result showed that, with the increase of H_2_O_2_ concentration, the cell viability of human hepatocyte LO2 cells decreased gradually (Figure 1(a)). When the concentration of H_2_O_2_ was 25 *μ*M, the cell viability of LO2 was significantly lower than that of blank control (without H_2_O_2_ treatment). When the concentration of H_2_O_2_ reached 100 *μ*M, the cell viability decreased to 50.17%. When the concentration of H_2_O_2_ reached 1000 *μ*M, the cell viability was only 29.35%. Therefore, the following experiments were carried out with H_2_O_2_ working solution of 100 *μ*M as the modeling agent.

#### 3.3.2. Determination of the Concentration Range of Polyphenols from Sweet Potato Leaves

Compared to blank control (without sweet potato leaf polyphenol treatment), the sweet potato leaf polyphenol treatment under the concentration range of 25–800 *μ*g/mL did not cause significant change of cell viability (Figure 1(b)). When the concentration of sweet potato leaf polyphenols reached 1000 *μ*g/mL, the cell viability of human hepatocyte LO2 cells decreased to 87.48%. With the further increase of sweet potato leaf polyphenol concentration, the cell viability was further reduced. Therefore, the following experiments were carried out with sweet potato leaf polyphenol working solution of 0–800 *μ*g/mL.

#### 3.3.3. Inhibition of Intracellular Reactive Oxygen Species of Total Polyphenols from Sweet Potato Leaves

**Figure 2:**
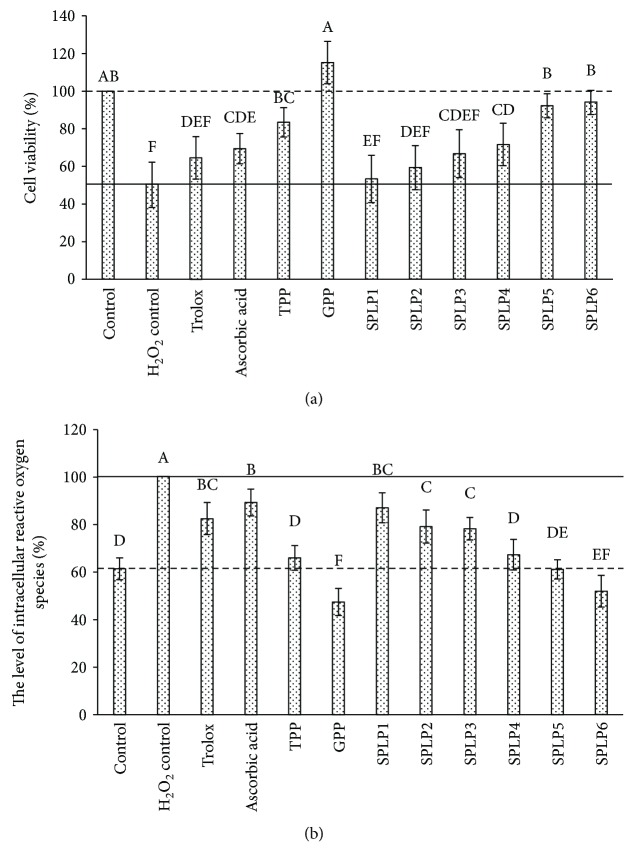
Protective effect of total polyphenols from sweet potato leaves on human hepatocyte LO2 oxidative stress. (a) The effect of total polyphenols from sweet potato leaves on the cell viability of oxidative stress LO2 cells. (b) The effect of total polyphenols from sweet potato leaves on the level of intracellular reactive oxygen species. Control was LO2 cells without H_2_O_2_ and antioxidant treatment; H_2_O_2_ control was the LO2 oxidative stress model group which was treated by H_2_O_2_ of 100 *μ*M; Trolox, ascorbic acid, TPP, and GPP were LO2 cells pretreated by 100 *μ*g/mL Trolox, ascorbic acid, tea polyphenols, and grape seed polyphenols, respectively, and then treated by 100 *μ*M H_2_O_2_; SPLP1, SPLP2, SPLP3, SPLP4, SPLP5, and SPLP6 were LO2 cells pretreated by sweet potato leaf polyphenols of 25, 50, 100, 200, 400, and 800 *μ*g/mL, respectively, and then treated by 100 *μ*M H_2_O_2_; the dashed lines represented the values of the blank control group, while the solid lines represented the values of the LO2 oxidative stress model group. Values were means ± SD of five determinations. The different letters above the different bars mean that the cell viability or the level of intracellular reactive oxygen species was significantly different (*p* < 0.05).

The effect of total polyphenols from sweet potato leaves on the cell viability of oxidative stress LO2 cells was shown in Figure 2(a). The total polyphenols from sweet potato leaves could significantly reduce the decline of LO2 cell viability induced by H_2_O_2_, and this effect possessed a dose-dependent manner. 25, 50, 100, 200, 400, and 800 *μ*g/mL sweet potato leaf polyphenol working solutions were able to restore the cell viability to 53.32%, 59.40%, 66.78%, 71.67%, 92.23%, and 94.01%, respectively. When the concentration of sweet potato leaf polyphenols reached 50 *μ*g/mL, the protective effect on LO2 cells was comparable to that of 100 *μ*g/mL Trolox and ascorbic acid. However, at the same concentration (100 *μ*g/mL), the protective effect of sweet potato leaf polyphenols, Trolox, ascorbic acid, and tea polyphenols showed no significant difference, which were all lower than that of grape seed polyphenols (the cell viability is 115.22%).

The effect of total polyphenols from sweet potato leaves on the level of intracellular reactive oxygen species was shown in Figure 2(b). Sweet potato leaf polyphenols decreased the level of intracellular reactive oxygen species significantly (*p* < 0.05) in a dose-dependent manner. When the concentration of sweet potato leaf polyphenols was 25 *μ*g/mL, the level of intracellular reactive oxygen species was decreased to 87.14% compared to the oxidative stress model group (100%), which was equivalent to 100 *μ*g/mL Trolox (82.51%) and ascorbic acid (89.30%). When the concentration of sweet potato leaf polyphenols was 50 and 100 *μ*g/mL, the level of intracellular reactive oxygen species was decreased to 79.19% and 78.32%, respectively, showing no significant difference compared to 100 *μ*g/mL Trolox. When the concentration of sweet potato leaf polyphenols was 200 and 400 *μ*g/mL, the level of intracellular reactive oxygen species was decreased to 67.34% and 61.17%, respectively, reaching the level of blank control and 100 *μ*g/mL tea polyphenols. When the concentration of sweet potato leaf polyphenols was 800 *μ*g/mL, the level of intracellular reactive oxygen species was decreased to 51.95%, which was equivalent to 100 *μ*g/mL grape seed polyphenols, and significantly lower than that of blank control.

#### 3.3.4. Inhibition of Intracellular Reactive Oxygen Species of Individual Phenolic Compounds from Sweet Potato Leaves

**Figure 3:**
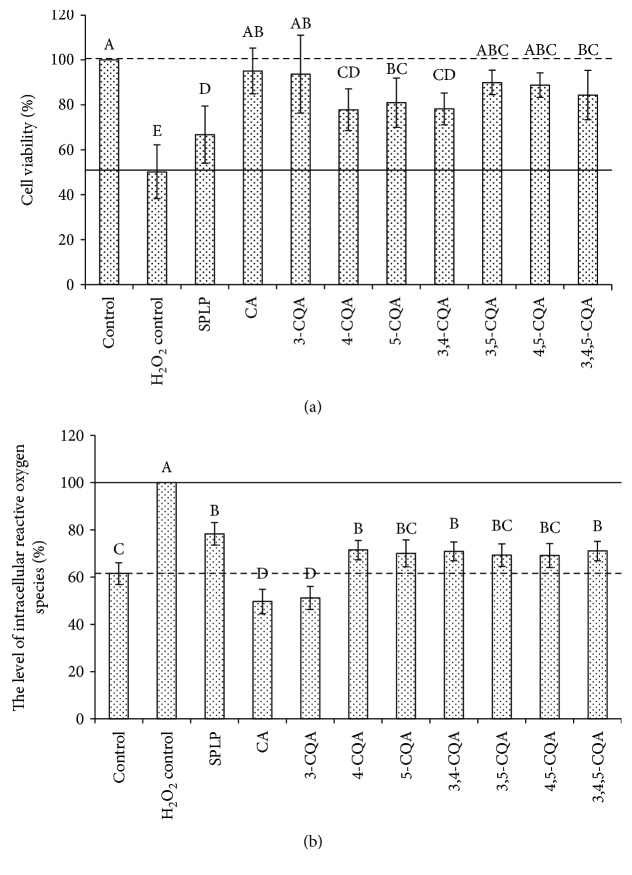
Protective effect of individual phenolic compounds from sweet potato leaves on human hepatocyte LO2 oxidative stress. (a) The effect of individual phenolic compounds from sweet potato leaves on the cell viability of oxidative stress LO2 cells. (b) The effect of individual phenolic compounds from sweet potato leaves on the level of intracellular reactive oxygen species. Control was LO2 cells without H_2_O_2_ and antioxidant treatment; H_2_O_2_ control was LO2 oxidative stress model group which was treated by H_2_O_2_ of 100 *μ*M; SPLP, CA, 3-CQA, 4-CQA, 5-CQA, 3,4-CQA, 3,5-CQA, 4,5-CQA, and 3,4,5-CQA were LO2 cells pretreated by 100 *μ*g/mL sweet potato leaf polyphenols, caffeic acid, 3-O-caffeoylquinic acid, 4-O-caffeoylquinic acid, 5-O-caffeoylquinic acid, 3,4-di-O-caffeoylquinic acid, 3,5-di-O-caffeoylquinic acid, 4,5-di-O-caffeoylquinic acid, 3,4,5-tri-O-caffeoylquinic acid, respectively, and then treated by 100 *μ*M H_2_O_2_; the dashed lines represented the values of the blank control group, while the solid lines represented the values of the LO2 oxidative stress model group. Values were means ± SD of five determinations. The different letters above the different bars mean that the cell viability or the level of intracellular reactive oxygen species was significantly different (*p* < 0.05).

The protective effect of individual phenolic compounds from sweet potato leaves on human hepatocyte LO2 oxidative stress was shown in Figure 3. Under the same sample concentration (100 *μ*g/mL), the cell viability of LO2 pretreated by caffeic acid and 3-O-caffeoylquinic acid was highest (95.12% and 93.71%, resp., but no significant difference), followed by 3,5-di-O-caffeoylquinic acid (89.96%), 4,5-di-O-caffeoylquinic acid (88.79%), 3,4,5-tri-O-caffeoylquinic acid (84.41%), 5-O-caffeoylquinic acid (80.97%), 3,4-di-O-caffeoylquinic acid (78.22%), 4-O-caffeoylquinic acid (77.83%), and the total polyphenols from sweet potato leaves (66.78%) (Figure 3(a)). Moreover, the cell viability of LO2 pretreated by all individual phenolic compounds and total polyphenols from sweet potato leaves was significantly higher than that of the LO2 oxidative stress model (50.17%) (*p* < 0.05), and the cell viability of LO2 pretreated by 3-O-caffeoylquinic acid, 3,5-di-O-caffeoylquinic acid, and 4,5-di-O-caffeoylquinic acid did not show a significant difference with that of blank control (100%).

The effect of individual phenolic compounds from sweet potato leaves on the level of intracellular reactive oxygen species was shown in Figure 3(b). All the tested samples decreased the level of intracellular reactive oxygen species significantly (*p* < 0.05) at the same concentration of 100 *μ*g/mL; especially caffeic acid and 3-O-caffeoylquinic acid, the intracellular reactive oxygen species level of which was decreased by 50.34% and 48.88% of H_2_O_2_ control, respectively, even lower than that of blank control. The intracellular reactive oxygen species level did not show a significant difference among 4-O-caffeoylquinic acid, 5-O-caffeoylquinic acid, 3,4-di-O-caffeoylquinic acid, 3,5-di-O-caffeoylquinic acid, 4,5-di-O-caffeoylquinic acid, 3,4,5-tri-O-caffeoylquinic acid, and total polyphenols from sweet potato leaves. However, considering the obvious difference in the content of individual phenolic compounds in sweet potato leaf polyphenols, it can be said that dicaffeoylquinic acids contribute most to the inhibition of intracellular reactive oxygen species of sweet potato leaf polyphenols.

The sequence of the effect of individual phenolic compounds from sweet potato leaves on the level of intracellular reactive oxygen species was not in accordance with that of the antioxidant activity, suggesting that there is no direct relationship between the antioxidant activity and protective effect on human hepatocyte LO2 oxidative stress. It has been reported that improving the endogenic ROS-regulating ability is the key to improve the ability of antioxidative stress [33]. By regulating the expression of oxidative stress-related genes, improving the endogenic ROS-regulating ability, intervening the signal transduction pathway of oxidative damage-inducing apoptosis, and protecting or repairing mitochondrial function, the cell damage induced by oxidative stress could be prevented effectively [33]. That is to say, the preventive effect of sweet potato leaf polyphenols on the oxidative stress of LO2 cells might be performed by regulating the expression of oxidative stress-related genes, rather than directly through the antioxidant effect, which needs to be verified by further study.

## 4. Conclusion

The antioxidant activity of sweet potato leaf polyphenols was significantly higher than those of ascorbic acid, tea polyphenols, and grape seed polyphenols. Among the individual phenolic compounds present in sweet potato leaves, caffeic acid showed the highest antioxidant activity, followed by mono- and dicaffeoylquinic acids and 3,4,5-tri-O-caffeoylquinic acid showed the lowest antioxidant activity. The intracellular ROS-inhibiting activity of sweet potato leaf polyphenols was similar to that of Trolox. The sequence of the inhibiting effect of individual phenolic compounds from sweet potato leaves on the level of intracellular ROS was not in accordance with that of the antioxidant activity, suggesting that there is no direct relationship between the antioxidant activity and protective effect on human hepatocyte LO2 oxidative stress. Sweet potato leaves could be a good source of biologically active polyphenols with multiple applications in the development of functional foods, health products, pharmaceuticals, and cosmetics. In pursuit of application in practical production, the biological activities of other compounds present in sweet potato leaves should also be investigated.

## Figures and Tables

**Table 1:** Individual phenolic compound composition of Yuzi No. 7 sweet potato leaf polyphenols.

Peak number	Retention time (min)	Identification^∗^	Standard curve	*R* ^2^	Peak area^a^	Content (%, DW)^a^
1	1.47	5-CQA	*y* = 11.372*x* − 0.428	0.9962	54.72 ± 0.85	2.42 ± 0.07
2	1.91	3-CQA	*y* = 9.909*x* + 0.286	1.0000	19.63 ± 0.23	0.98 ± 0.02
3	2.10	4-CQA	*y* = 25.894*x* − 17.128	0.9988	32.27 ± 0.19	0.95 ± 0.01
4	2.92	CA	*y* = 28.183*x* − 1.211	1.0000	4.12 ± 0.07	0.09 ± 0.01
5	4.16	4,5-CQA	*y* = 9.208*x* − 7.244	0.9987	386.51 ± 3.68	21.38 ± 0.21
6	4.54	3,5-CQA	*y* = 18.056*x* − 18.405	0.9981	1292.36 ± 22.32	36.30 ± 0.19
7	4.88	3,4-CQA	*y* = 15.353*x* − 12.021	0.9987	371.93 ± 5.16	25.01 ± 0.42
8	6.87	3,4,5-CQA	*y* = 6.218*x* − 5.158	0.9949	26.84 ± 0.99	2.57 ± 0.08

^∗^5-CQA: 5-O-caffeoylquinic acid; 3-CQA: 3-O-caffeoylquinic acid; 4-CQA: 4-O-caffeoylquinic acid; CA: caffeic acid; 4,5-CQA: 4,5-di-O-caffeoylquinic acid; 3,5-CQA: 3,5-di-O-caffeoylquinic acid; 3,4-CQA: 3,4-di-O-caffeoylquinic acid; and 3,4,5-CQA: 3,4,5-tri-O-caffeoylquinic acid. ^a^Values were means ± SD of three determinations.

**Table 2:** Antioxidant activity of Yuzi No. 7 sweet potato leaf polyphenols, tea polyphenols, and grape seed polyphenols.

Samples^∗^	Sample concentration (*μ*g/mL)					
	5	10	20	5	10	20
	·O_2_^−^ scavenging activity (*μ*g·ACE/mL)			Oxygen radical absorbance capacity (*μ*g·TE/mL)		
SPLP	14.57 ± 0.31^a^	30.56 ± 2.59^a^	62.71 ± 2.99^a^	22.35 ± 1.59^a^	33.72 ± 2.61^a^	55.68 ± 1.45^a^
TPP	3.60 ± 0.28^b^	7.29 ± 0.31^b^	10.62 ± 0.45^b^	16.67 ± 2.98^b^	32.23 ± 1.22^a^	43.53 ± 0.59^b^
GPP	3.02 ± 0.11^c^	3.18 ± 0.42^c^	6.73 ± 0.12^c^	13.75 ± 0.62^b^	29.21 ± 1.68^b^	43.54 ± 0.77^b^

^∗^SPLP: total polyphenols from sweet potato leaves; TPP: total polyphenols from tea; GPP: total polyphenols from grape seeds. ^a–c^Data in the same column that were significantly different were represented by different letters (*p* < 0.05).

**Table 3:** Antioxidant activity of individual phenolic compounds from sweet potato leaves.

Samples^∗^	·O_2_^−^ scavenging activity (*μ*g·ACE/mL)	Oxygen radical absorbance capacity (*μ*g·TE/mL)
SPLP	30.56 ± 2.59^b^	33.72 ± 2.61^c^
CA	51.12 ± 5.35^a^	56.78 ± 4.12^a^
3-CQA	22.97 ± 2.81^c^	41.23 ± 1.06^b^
4-CQA	19.36 ± 1.45^c^	39.15 ± 1.58^bc^
5-CQA	20.12 ± 2.79^c^	42.58 ± 3.66^b^
3,4-CQA	20.68 ± 1.55^c^	39.91 ± 8.37^bc^
3,5-CQA	21.69 ± 1.42^c^	35.21 ± 2.11^bc^
4,5-CQA	22.14 ± 2.15^c^	42.16 ± 3.89^b^
3,4,5-CQA	15.03 ± 1.12^d^	32.21 ± 1.62^c^

^∗^The concentration of all tested samples was 10 *μ*g/mL; SPLP: total polyphenols from sweet potato leaves; CA: caffeic acid; 3-CQA: 3-O-caffeoylquinic acid; 4-CQA: 4-O-caffeoylquinic acid;5-CQA: 5-O-caffeoylquinic acid; 3,4-CQA: 3,4-di-O-caffeoylquinic acid; 3,5-CQA: 3,5-di-O-caffeoylquinic acid; 4,5-CQA: 4,5-di-O-caffeoylquinic acid; 3,4,5-CQA: 3,4,5-tri-O-caffeoylquinic acid. ^a–d^Data in the same column that were significantly different were represented by different letters (*p* < 0.05).
